# The risk of malignancy in patients with IgG4-related disease: a systematic review and meta-analysis

**DOI:** 10.1186/s13075-021-02652-2

**Published:** 2022-01-05

**Authors:** Tingfeng Yu, Yaxian Wu, Jia Liu, Yanyan Zhuang, Xiaoyan Jin, Lingyun Wang

**Affiliations:** 1 Department of Gastroenterology, Sun Yat-sen Memorial Hospital, Guangzhou, China; 2 Department of General Practice, Sun Yat-sen Memorial Hospital, Guangzhou, China

**Keywords:** IgG4-RD, Malignancy, SIR, Meta-analysis

## Abstract

**Background:**

The relationship between IgG4-related disease (IgG4-RD) and the risk of malignancy is still controversial. This article focused on assessing the risk of cancer in patients with IgG4-RD by meta-analysis.

**Methods:**

We conducted a systematic review of the literature and meta-analysis characterizing the associated risk of overall malignancy and four site-specific malignancies (pancreas, lung, gastric and lymphoma) in patients with IgG4-RD. A search from 2003 to 2020 was performed using specified terms from PubMed, Embase, Web of Science and SinoMed. Random-effects model analysis was used to pool standardized incidence ratios (SIRs) and 95% confidence intervals (CIs). Subgroup and sensitivity analyses were conducted to clarify the heterogeneity of the included studies. Begg’s funnel plot and Egger’s linear regression test were used to evaluate the bias of the meta-analysis. A *P* value < 0.05 indicated the existence of publication bias.

**Results:**

A total of 10 studies were included in the article. The overall SIR estimates suggested an increased risk of overall cancer in IgG4-RD patients (SIR 2.57 95% CI 1.72–3.84) compared with the general population. The specific SIRs for pancreas and lymphoma were higher than those of the general population in IgG4-RD patients (SIR 4.07 95% CI 1.04–15.92, SIR 69.17 95% CI 3.91–1223.04, respectively). No significant associations were revealed in respiratory and gastric cancer (SIR 2.14 95% CI 0.97–4.75, SIR 0.95 95% CI 0.24–3.95, respectively). Four studies were found to be the major sources of heterogeneity by sensitivity analysis. There was no evidence of publication bias via Egger’s test.

**Conclusion:**

Compared with the general population, patients with IgG4-RD appear to have a higher risk of overall cancer, especially pancreatic and lymphoma. The risk of lung and gastric cancer was not different between IgG4-RD patients and the general population.

**Supplementary Information:**

The online version contains supplementary material available at 10.1186/s13075-021-02652-2.

## Introduction

IgG4-related disease (IgG4-RD), featuring dense lymphoplasmacytic IgG4+ plasma cells, storiform fibrosis and obliterative phlebitis in histopathology, is a chronic fibroinflammatory autoimmune disease with multisystemic involvement. It is crucial to differentiate malignant disease from IgG4-RD for the application of glucocorticoids, the first-line treatment for IgG4-RD.

The risk of malignancies in patients with IgG4-RD has not been clarified. There are differing opinions on this issue. It should be noted that with ongoing further research, the initiation of immunomodulatory treatment and biologic agents has a potential therapeutic risk of increasing the incidence of malignancy by altering the normal function of immunosurveillance in addition to acting as an effective therapy in IgG4-RD patients [1, 2]. Thus, understanding the baseline risk of malignancy is of great importance in patients with IgG4-RD.

To date, there has been no meta-analysis evaluating the risk of malignancy in IgG4-RD. In this article, we review the reported data on the incidence of malignancy and perform a systematic review and meta-analysis to assess the risk of malignant disease.

## Materials and methods

### Search strategy and selection criteria

To identify studies characterizing the risk of malignancy in patients with IgG4-RD compared with the general population, a search of PubMed, Embase, Web of Science and SinoMed for literature in English published between 2003 and 2020 was conducted using the following specified search terms: “IgG4-Associated Autoimmune Disease”, “IgG4-Related Disease”, “Autoimmune related systemic disease”, “IgG4 Related Disease” and “Autoimmune Pancreatitis” combined with “Cancer”, “Tumor”, “Malignancy” and “Carcinoma”. The inclusion criteria were as follows: (1) observational-type study design (including prospective, retrospective, database, cohort and case-control studies); (2) more than 100 patients; (3) standardized incidence ratio (SIR) and 95% confidence interval (CI) reported or calculated with information from the literature; and (4) the diagnosis of IgG4-RD was in accordance with the comprehensive diagnostic criteria or fulfilled the specific organ diagnostic criteria. Cancer outcomes, diagnosed concurrently or after the diagnosis of IgG4-RD, were assessed using medical records and reliable radiographic or histological findings. The exclusion criteria were as follows: (1) reviews, case reports, and conference abstracts and (2) a lack of overall SIR and confidence intervals or ambiguous data that were unavailable for calculating the SIR.

### Data extraction and quality assessment of the studies

Two investigators independently assessed the quality of the selected studies and extracted the original literature data, including author, country, calendar period, study type, comparison population, number of patients with IgG4-RD, observed and expected cancer cases, diagnostic criteria, adjusted factors, length of follow-up, SIR and 95% CI data. The Newcastle-Ottawa Scale (NOS) was applied to evaluate the quality of the study, where a score of 5 or less was classified as low quality. Disagreements were resolved through discussion and consensus and if necessary, turning to the third investigator for a final decision.

### Statistical analysis

The association between IgG4-RD and various cancers was analysed based on available data, and the pooled SIRs with 95% CIs were applied to evaluate its efficacy. SIRs with 95% CIs for overall cancer and organ-specific cancer risks were pooled only when data from two or more studies were available. In situations in which SIRs were not reported, they were calculated from the numbers of observed and expected malignancies (SIR = number of observed malignancies per number of expected malignancies), and the 95% CI was determined assuming that the frequency of observed cases followed a Poisson distribution. Heterogeneity was assessed by means of the chi-square test and *I*^2^ test. *I*^2^ values were used to quantify heterogeneity at three levels: low (<50%), moderate (50–75%), and high (≥ 75%). If significant heterogeneity (*P*<0.05 or *I*^2^>50%), the random-effects model was applied. Otherwise, the fixed-effects model was utilized. Subgroup and sensitivity analyses were performed to explore the potential sources of heterogeneity. Publication bias was assessed via Begg’s funnel plot and Egger’s linear regression test. All statistical analyses were performed using Stata 12.0 software.

## Results

### Characteristics of the included studies

**Fig. 1:**
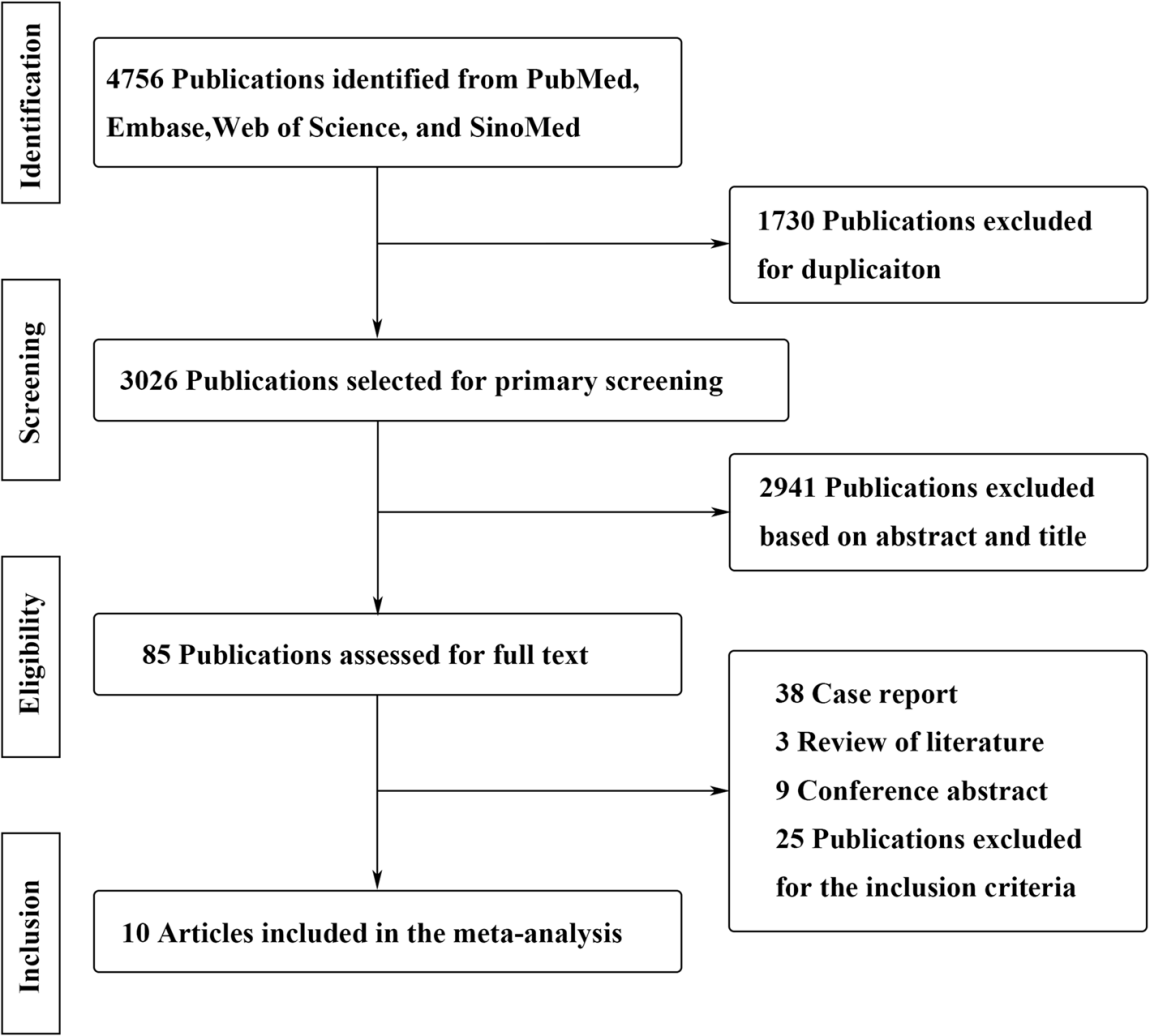
A flow diagram showing article selecting

A total of 4756 articles were identified according to the search criteria, and 1730 publications were excluded for duplication. A total of 2941 studies were excluded based on the abstract and title. Through full-text evaluations, a total of 10 studies met the inclusion criteria. A flow diagram for the retrieval and inclusion of studies is shown in Fig. 1. The basic research characteristics of the article are shown in Table 1. The number of patients in the included cohort studies from different countries between 1986 and 2018 ranged from 106 to 587. Among the 10 included articles, 6 studies were screened as high quality (NOS score ≥ 6).

**Table 1:** Characteristics of the included studies

Reference	NOS	Country	Calendar period	Study type	Comparison population	Total no. of patients with IgG4-RD	Observed cancer cases	Expected cancer cases	Diagnostic criteria for IgG4-RD	Adjusted factors	Follow-up	Overall cancer SIR(95% CI)
Takahashi [6]	6	America	1986–2008	Registry-based	General America population	111	3.00	NA	HISORt criteria	Age, sex	331 person-years	16.0 (3.3–45.5)
Yamamoto et al. [8]	5	Japan	1997–2011	Registry-based	General Japan population	106	6.00	2.87	CDC	Age, sex	3.1 years	2.09 (0.77,4.55)
Shiokawa et al. [7]	7	Japan	2001–2011	Multicentre cohort	General Japan population	108	18.00	6.70	Asian diagnostic criteria [44], HISORt criteria	Age, sex, calendar year	3.3 years/415.7 person-years	2.70 (1.40,3.90)
Hirano et al. [3]	6	Japan	1997–2012	Multicentre cohort	General Japan population	113	15.00	NA	ICDC, CDC	Age, sex	6.1 years	1.04 (0.57,1.75)
Huggett et al. [9]	5	UK	2004–2013	Double-center cohort	General UK population	115	9.00	4.50	ICDC	Age, sex	2.7 years	2.00 (0.91,3.80)
Inoue et al. [10]	5	Japan	2005–2013	Registry-based	General Japan population	235	15.00	12.90	IgG4-RD Pathology Consensus Statement [45], ICDC	NA	3.1 years	1.16 (0.70,1.93)
Asano et al. [4]	6	Japan	1992–2012	Multicentre cohort	General Japan population	158	34.00	16.9	CDC, ICDC	Age, sex, calendar year	6.0 years/940 person-years	2.01 (1.34,2.69)
Sekiguchi et al. [12]	4	America	1994–2012	Registry-based	General America population	166	9.0	NA	Modified CDC	NA	2.4 years	4.50 (1.50,7.50)
Ahn et al. [5]	6	Korea	2006–2015	Single-centre cohort	General Korea population	118	5.0	0.52	CDC	NA	0.7 years	9.61 (3.12,22.40)
Tang et al. [13]	6	China	2011–2018	Single-centre cohort	General China population	587	10.0	NA	CDC	Age, sex	5.1 years	2.78 (1.33,5.12)

*SIR* standardized incidence ratio, *CI* confidence interval, *NOS* The Newcastle-Ottawa Scale, *CDC* Comprehensive Diagnostic Criteria for IgG4-RD [46], *ICDC* International Consensus Diagnostic Criteria for Autoimmune Pancreatitis [47], *HISORt Criteria* Diagnostic Criteria for Autoimmune Pancreatitis (Including Histology, Pancreatic imaging, Serology, Other organ involvement, Response to steroid therapy) [48], *NA* not available

### Meta-analysis results

All the studies reported the overall SIRs for the malignancy. Only 4 articles [3–6] calculated the SIR of specific-organ malignancy, and 3 studies [3, 4, 7] reported the SIR before or after 1 year of the diagnosis of IgG4-RD. In total, 3 articles [5, 8, 9] needed recalculation of the overall risk of cancer.

**Fig. 2:**
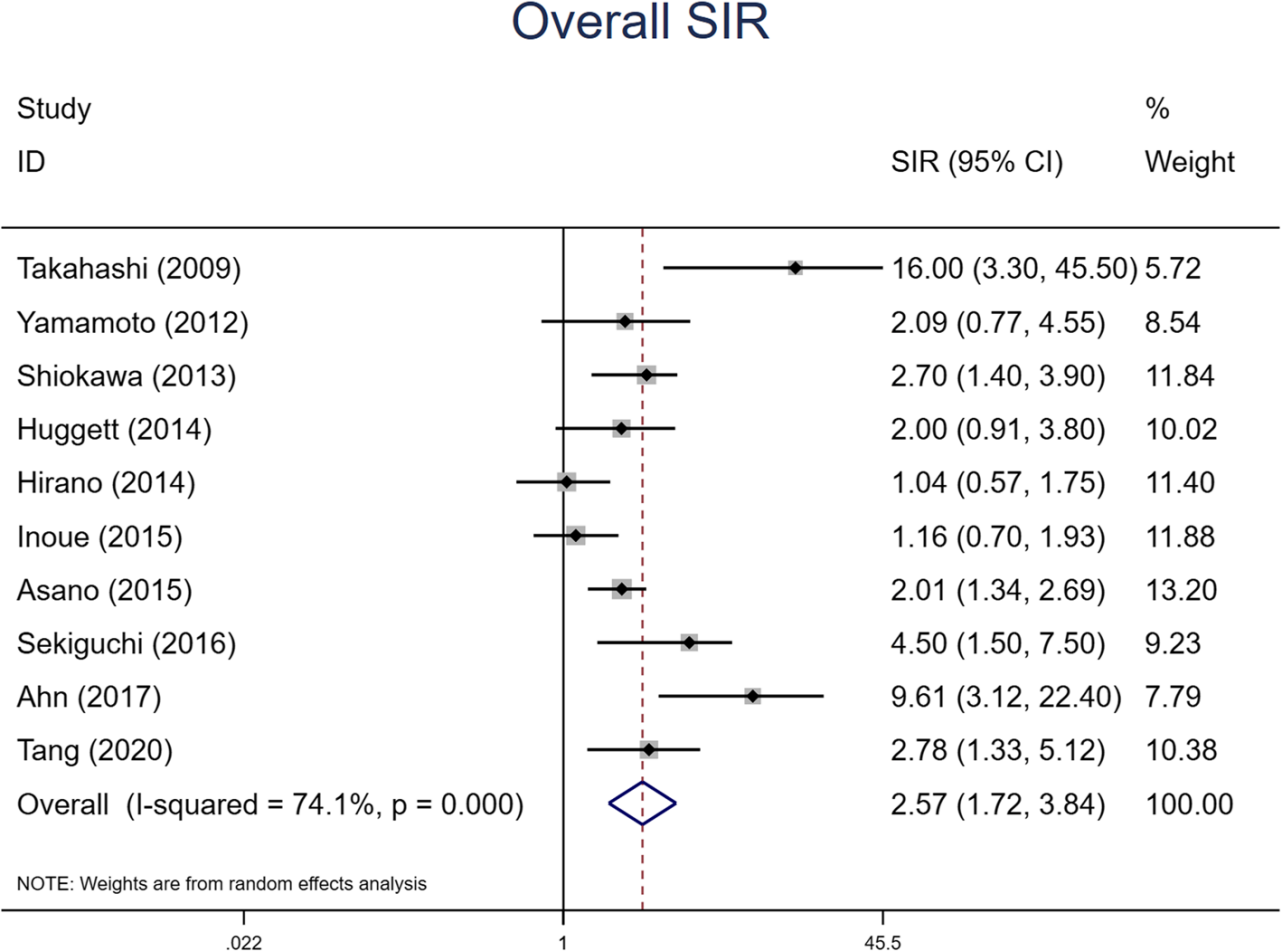
Forest plot of IgG4-RD associated with overall characteristics

**Fig. 3:**
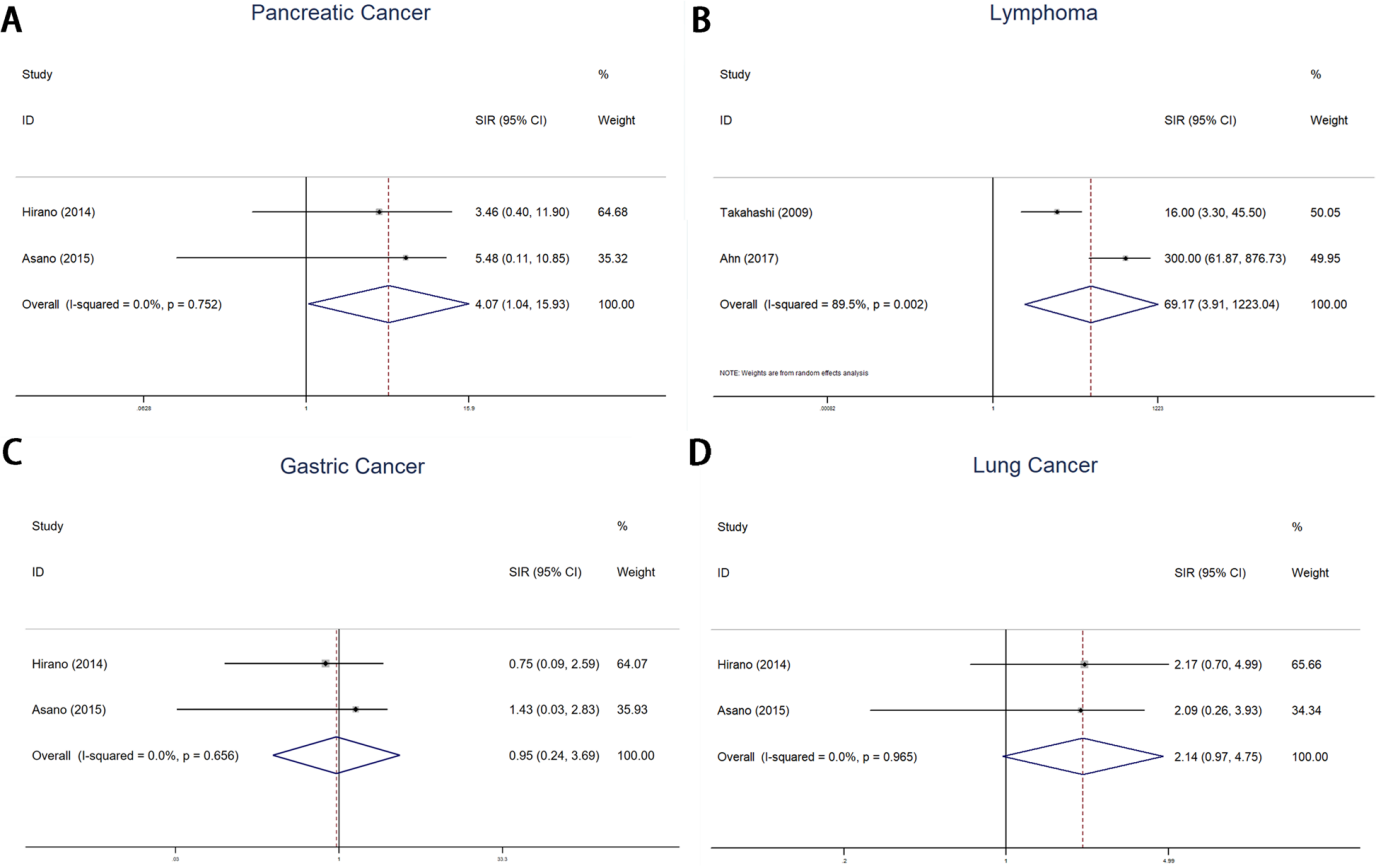
Forest plots of IgG4-RD associated with specific site cancer. **a** Pancreatic Cancer; **b** Lymphoma; **c** Gastric Cancer; **d** Lung Cancer

**Fig. 4:**
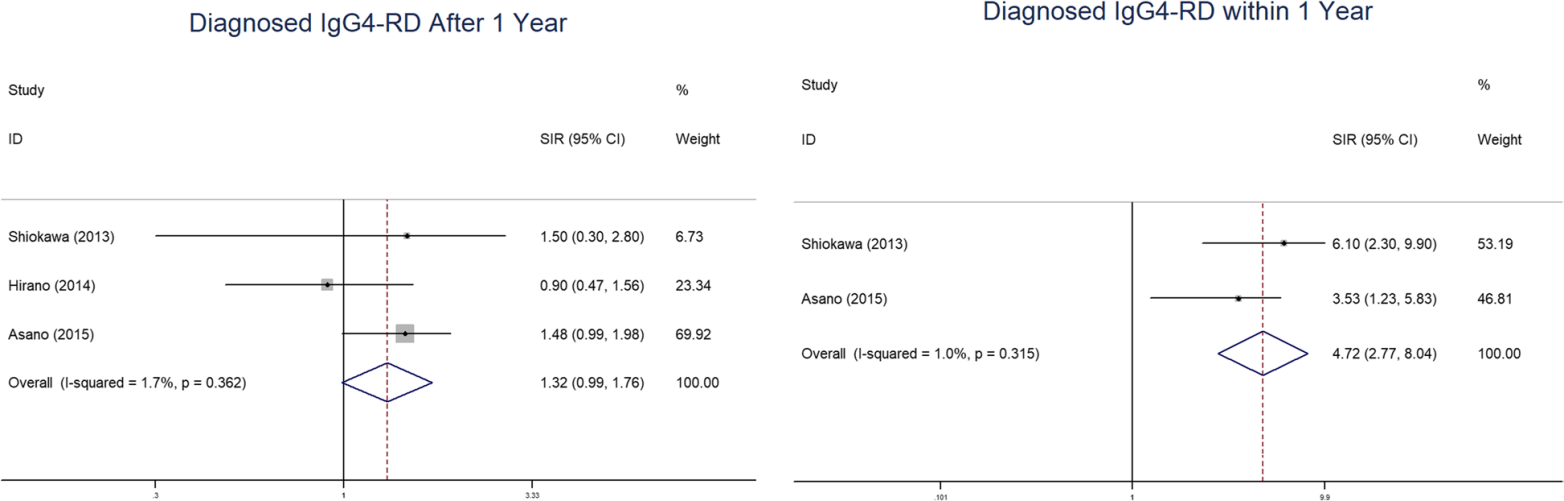
Forest plots of diagnosed IgG4-RD after or within 1 year

Patients with IgG4-RD had an increased risk of malignancy (SIR = 2.57, 95% CI 1.72–3.84). A forest plot is shown in Fig. 2. In view of the moderate heterogeneity, a random-effects model was performed (*I*^2^ = 74.1%, *P* <0.01). Significant increases were observed in the risk of cancer of the pancreas (SIR 4.07 95% CI 1.04–15.92, Fig. 3A) and lymphoma (SIR 69.17 95% CI 3.91–1223.04, Fig. 3B). No higher levels of risk of gastric cancer (SIR 0.95 95% CI 0.24– 3.95, Fig. 3C) or lung cancer (SIR 2.14 95% CI 0.97–4.75, Fig. 3D) were discovered in the study. Obvious increases were found in the pooled SIR within 1 year of IgG4-RD diagnosis (SIR 4.72 95% CI 2.77–8.04), while such an association was not found after 1 year of diagnosis (SIR 1.32 95% CI 0.99–1.76) Figs. 4.

**Fig. 5:**
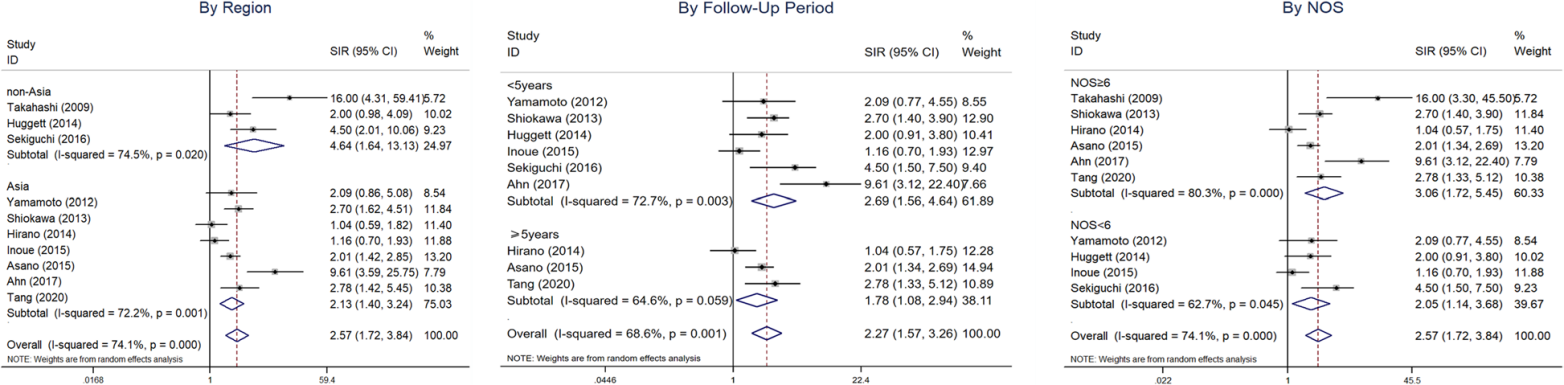
Forest plots of subgroup analysis

To further understand the source of heterogeneity, a subgroup analysis (Table 2, Fig. 5) was conducted by study quality, geographic region and mean follow-up time. Stratification based on study quality resulted in a pooled SIR of 3.06 (95% CI 1.72–5.45) for the studies with NOS≥6 and 2.05 (95% CI 1.14–3.68) for the rest of the studies. Stratification according to geographic region revealed that the pooled SIR of Asia was 2.13 (95% CI 1.40–3.24), while that of non-Asia was 4.64 (95% CI 1.64–13.13). Stratified by the follow-up time, the pooled SIR for those <5 years was 2.69 (95% CI 1.56–4.64), and the other was 1.78 (95% CI 1.08–2.94). When the studies by Takahashi et al. [6], Ahn et al. [5], Hirano et al. [3] and Inoue et al. [10] were excluded, the *I*^2^ value was reduced to 0% with a *P* value > 0.05, which strongly indicated the heterogeneity resulting from these studies.

**Table 2:** Subgroup analysis of overall cancer risk in the combined IgG4-RD population

Subgroup	No. of studies	SIR (95% CI)	Heterogeneity analysis	
			*I*^2^	*p* value
Study quality				
NOS<6	4	2.05 (1.14–3.68)	62.7%	*P*<0.05
NOS≥6	6	3.06 (1.72–5.45)	80.3%	*P*<0.05
Geographic region				
Asia	7	2.13 (1.40–3.24)	72.2%	*P*<0.05
Non-Asia	3	4.64 (1.64–13.13)	74.5%	*P*<0.05
Follow-up time, years				
<5	6	2.69 (1.56–4.64)	72.7%	*P*<0.05
≥5	3	1.78 (1.08–2.94)	64.6%	*P*>0.05

A sensitivity analysis was performed to assess the stability of the meta-analysis of the overall risk of malignancy. When any single study was excluded, the corresponding pooled SIRs were not substantially altered. Statistically similar results indicated the stability of the meta-analysis. The *P* value for Begg’s test was 0.049, and the *P* value for Egger’s test was 0.056 (>0.05). In view of the numbers of included studies, the statistical performance was more sensitive in the latter [11]. Egger’s publication bias plot for the overall risk of malignancy in IgG4-RD patients was symmetric in appearance with a *P* value >0.05, which meant that there was no evidence of publication bias in the meta-analysis.

## Discussion

Our meta-analysis revealed an association between IgG4-RD and increased cancer risk compared with the general population. The outcome successfully shed light on IgG4-RD increasing the risks of overall cancer, particularly of cancer of the pancreas and lymphoma. In addition, no significant associations were discovered between IgG4-RD and the lung or stomach. Four studies [3, 8–10] lacked evidence to support our results, and the rest of the included articles yielded results similar to ours [4–9, 12, 13].

We detected a significantly increased risk of pancreatic cancer in IgG4-RD patients compared with the general population. Generally, chronic pancreatitis is a risk factor for developing pancreatic cancer [14]. It is biologically plausible that autoimmune pancreatitis is speculated to be associated with a high risk of developing cancer. Type 1 AIP was classified as the manifestation of IgG4-RD [15]. Inoue et al. [16] reported a case of AIP with pancreatic cancer, which had an increase in serum IgG4. In line with our results, a study [17] from Germany observed 6 malignant diseases in 5 patients with AIP, and the overall cancer SIR was 17.3 (95% CI 5.9–35.8). Another study from Japan conducted by Shimizu et al. showed that there were no obvious correlations between AIP and malignancy, and the SIR of the study was 2.14 (95% CI 0.74–3.54) [18]. Such a difference might be partially explained by the diminutive sample sizes and the lack of sufficient statistical power in their studies. Although Gutpa et al. [19] discovered that preneoplastic ductal lesions were frequently prevalent in AIP patients, the data regarding AIP with pancreatic cancer in situ were poor. Kamisawa et al. showed that K-ras gene mutations occurred in the gastrointestinal mucosa of AIP patients, implying that AIP is a risk factor for gastric and colonic cancer [20]. It should be noted that a matched-control study conducted by Hart et al. [21] did not agree with ours, with a hazard ratio of 0.64 (95% CI 0.27–1.51).

Lung cancer complicated with IgG4-RD has been reported in recent years [22, 23]. The incidence of lung nodules was high in IgG4-RD patients [24]. Fujimoto et al. [25] found >20 IgG4+ plasma cells per high-power field from patients who underwent surgical resection in a minority of non-small-cell lung cancer patients, some of whom had an IgG4/IgG ratio >40%. Matsui et al. [26] concluded that the incidence of malignancies was higher in IgG4-RD patients than in the general population, given that the SIR for malignancies was 2.85 (95% CI 1.24–4.46), supporting an increased risk of lung cancer in IgG4-RD patients. However, no significant association was revealed between respiratory cancer and IgG4-RD in the meta-analysis. The analysed studies here were not adjusted for some important risk factors, such as smoking, an important risk factor for lung cancer.

It is also interesting that the pooled SIR for gastric cancer in IgG4-RD did not show an increased risk compared with the general population. However, in the study of Shiokawa et al., gastric cancers accounted for the highest proportion among all IgG4-RD-CA patients. In a meta-analysis focusing on the risk of gastric cancer in autoimmune diseases, IgG4-RD was associated with an increased risk of gastric cancer, with a relative risk of 1.69 (95% CI 1.00–2.87) [27]. *Helicobacter pylori* infection might increase the risk of developing gastric cancer via the immune cross-response in IgG4-RD patients [28]. The relationship between IgG4-RD and gastric cancer needs to be clarified in the future.

The increased risk of nonsolid tumours, such as lymphoma and leukaemia, was also a large part of the investigation among IgG4-RD-CA patients. Our meta-analysis presented a significant association between lymphoma and IgG4-RD. However, the high heterogeneity of the combined SIR reminded us to interpret the results with caution (*I*^2^ = 89.5%, *P* <0.01). Previous studies suggested that the clonal expansion of IgG4+ plasma cells against a background of IgG4-RD chronic inflammation may participate in the progression of mucosa-associated lymphoid tissue (MALT) production [29, 30]. A study [31] from Poland revealed that some patients with immunohistochemical diagnosis of orbital MALT lymphoma were consistent with the criteria of IgG4-RD. Guo et al. [32] and Hirano et al. [3] reported IgG4-RD concomitant with leukaemia. Ichiki et al. [33] showed that an increased number of IgG4+ cells was confirmed in the bone marrow.

The concrete mechanism between IgG4-RD and malignant disease is still inconclusive. The possible hypotheses include chronic inflammatory stimulation and dysfunction of the immune system. Inflammation increases the risk of cancer and has been elucidated in several diseases [14]. T helper type 2 (Th2) cells and regulatory cells (Tregs) are increased in IgG4-RD patients. IL-4 and IL-13, secreted by activated Th2 cells, induce aberrant expression of activation-induced cytidine deaminase (AID), which can result in DNA mutations with potential cancer development. Transforming growth factor-β (TGF-β), a cytokine produced by Tregs, has been verified to regulate cancer progression in different signalling pathways [14, 34, 35]. IL-33 may also play a role in the development of malignancy through IL-33/ST2 in IgG4-RD [36, 37]. Impairment of the immune system might also participate in the tumour progression of IgG4-RD. M2-polarized tumour-associated macrophages, which secrete cytokine factors such as IL-10 and TGF-β to support immunosuppression in tumours, have been linearly correlated with IgG4+ plasma cells in pancreatic cancer [38, 39]. The Th2-biased inflammatory response promotes the production of IgG4, which may inhibit effector cell functions against tumours. For example, IgG4 can antagonize IgG1-mediated antimelanoma immunity through competition with IgG1 for FcγR binding, indirectly contributing to tumour growth [40, 41]. Notably, there is a different view on the relationship between cancer and IgG4-RD. Wallace et al. [42] reasoned that neoantigens induced by cancer trigger a series of autoimmune responses, implying that IgG4-RD could subsequently develop after cancer. Early evidence proved that tumour antigens could elicit the autoimmune response, promoting the development of scleroderma in patients with cancer gene mutations [43]. This idea was supported by Shiokawa et al., who considered that the performance of IgG4-RD was a possibly paraneoplastic syndrome. A certain portion of IgG4-RD patients did not relapse or had resolution after the successful treatment of their cancer [7]. In our meta-analysis, the cancer risk of SIR in IgG4-RD diagnosed within 1 year was significantly higher, which was in accordance with the paraneoplastic syndrome assumption. Another possibility was the incidental diagnosis of cancer following extensive investigations after the diagnosis of IgG4-RD, which led to the difference in SIR within 1 year.

Subgroup analysis failed to clearly determine the possible source of heterogeneity, while the sensitivity analysis discovered that four articles were responsible for the heterogeneity of the meta-analysis. After further analysing these articles, we considered that the heterogeneity may be explained by the following study limitations. First, the follow-up period of the study from Korea [5] was only 0.7 years, which was too short to evaluate the real incidence of cancer. Second, Hirano et al. [3] excluded patients diagnosed concurrently with IgG4-RD and malignant diseases from their study. Third, the heterogeneity of the study from Inoue et al. [10] might be due to a retrospective search of the radiology database.

The primary advantage of this study was that we used the SIR to evaluate the relative risk of IgG4-RD compared with the general population, which deserved more attention for the diagnosis and management of IgG4-RD patients. Furthermore, pancreatic cancer and lymphoma were found to be more frequently seen in IgG4-RD-CA patients. However, the high heterogeneity in the combined SIR of lymphoma indicated that it should be interpreted with caution.

However, the limitations of our study are obvious. First, most of the included studies were retrospectively retrieved from records or national databases, the potential deviation could not be ignored, and there was moderate heterogeneity among the included studies. However, from the results of the sensitivity analysis, the heterogeneity had no substantial influence on the overall pooled SIR. Second, the confounding factors were not fully standardized, especially in terms of the incidence of organ-specific cancer. Third, more included studies and larger sample sizes are needed to improve the meta-analysis results. Fourth, most of the follow-up periods in the included studies were insufficient to fully assess the risk of cancer. Fifth, the lack of uniform diagnostic criteria for IgG4-RD in the included studies weakened the statistical effect. Sixth, not all articles reported organ-specific cancer SIRs.

## Conclusions

Taken together, IgG4-RD increases the risk of malignancy compared with the general population. Pancreatic cancer and lymphoma are associated with a high incidence rate in IgG4-RD patients according to the present data. Therefore, it is of great importance to screen for cancer in the management and diagnosis of IgG4-RD. However, we should be cautious when interpreting the results since most of the present studies are observational and the follow-up periods are relatively short for completely assessing the incidence rate of cancer. The concrete mechanism of IgG4-RD in developing malignant disease is unclear. Further higher-quality studies are needed to verify and elucidate the relationship between IgG4-RD and tumours.

## Supplementary Information

**Additional file 1.**

**Additional file 2.**

## Data Availability

Not applicable.

## Abbreviations

IgG4-RD: IgG4-related disease
SIR: Standardized incidence ratio
CI: Confidence interval
NOS: The Newcastle-Ottawa Scale
MALT: Mucosa-associated lymphoid tissue
IgG4-RD-CA: IgG4-related disease with cancer
