# Supplementary figures and images for: The risk of malignancy in patients with IgG4-related disease: a systematic review and meta-analysis
Source: Arthritis Res Ther. 2022 Jan 5;24:14. doi: 10.1186/s13075-021-02652-2 (PMC8728936; doi:10.1186/s13075-021-02652-2)

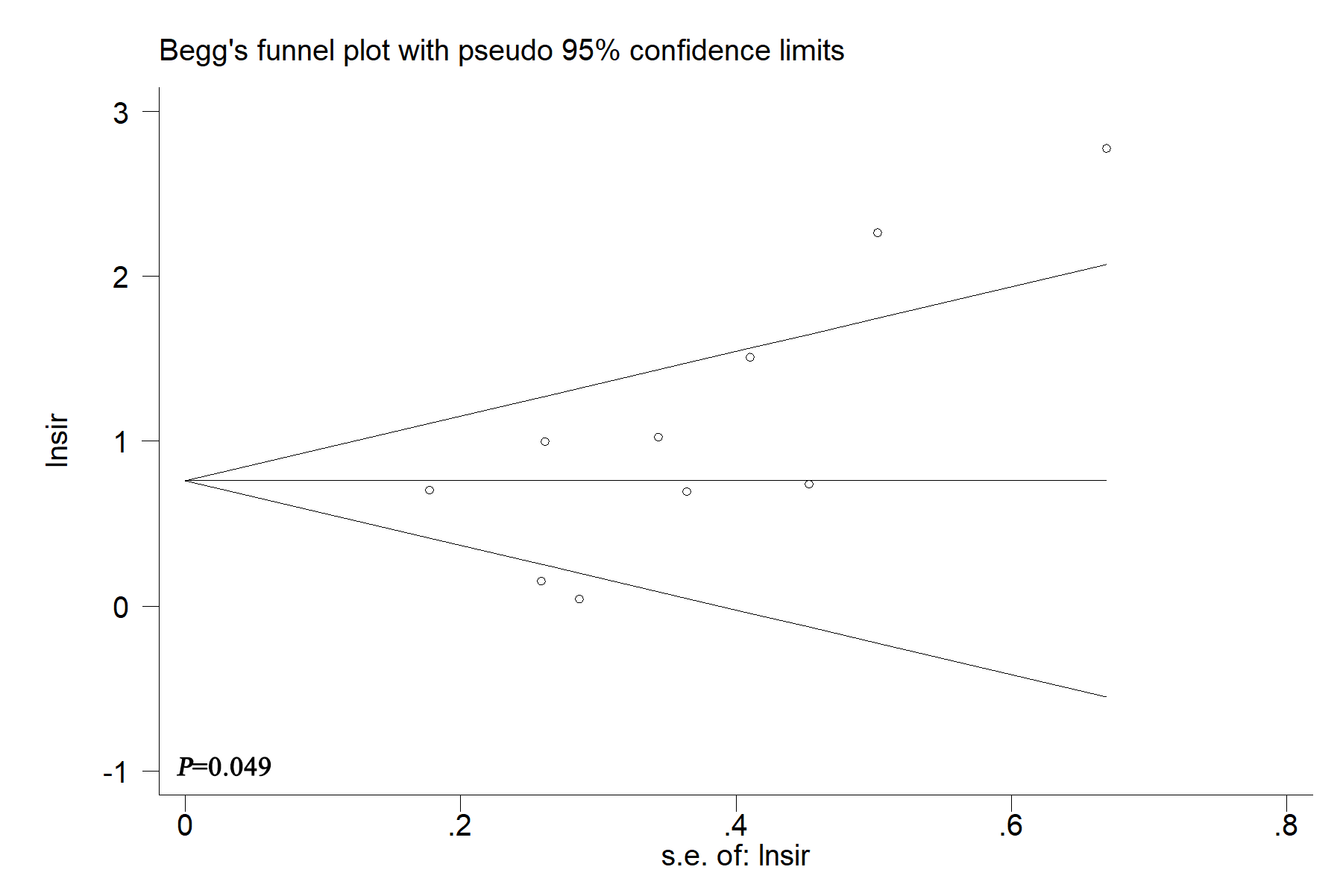

Supplement: Supplementary file 1 — Additional file 1. [file 13075_2021_2652_MOESM1_ESM.png]

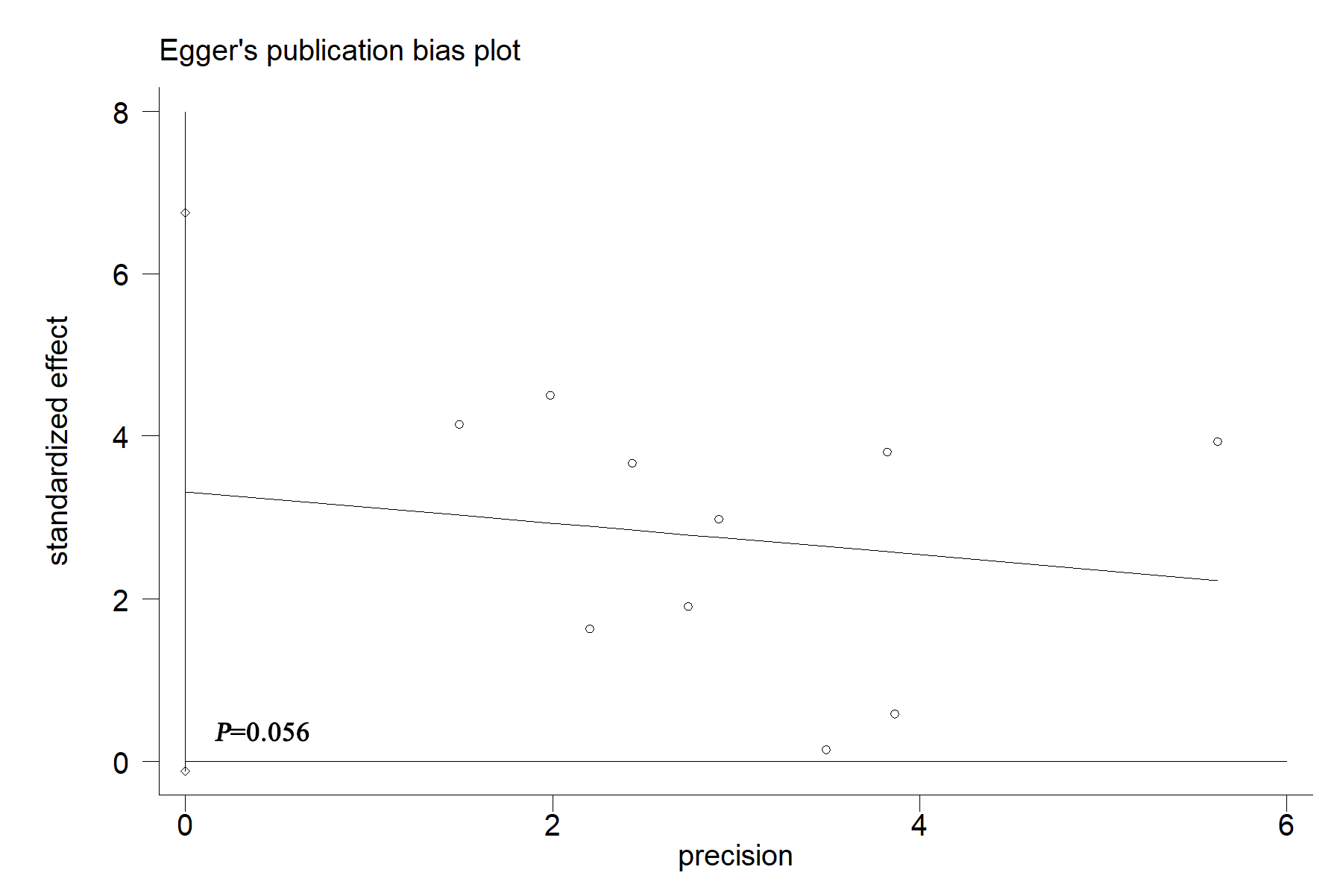

Supplement: Supplementary file 2 — Additional file 2. [file 13075_2021_2652_MOESM2_ESM.png]
